# The Whiskey Cure

**Published:** 1870-12

**Authors:** 


### THE WHISKEY CURE.

Whiskey cures a great many ailments, infallibly, by killing the patient. Many years ago we fell into conversation with a gentleman on board a Mississippi steamer on the subject of curing consumption by drinking whiskey largely. Day after day he would narrate instances of almost miraculous recovery from low states of disease, from apparent desperate bodily conditions. After letting him run out, replying not a word, wanting only his facts, we began at no one, his own wife had asked to know the present condition of each of the cured. His wife had died of——; Mr. A. had died too of——; Mr. B. did well, and had he not had an attack of———would no doubt have been a well man to this day—and so with every single case he had narrated; the amount of it all was, every one had got well of consumption, but had died of something else. It will be useful to the reader, in many cases at least, when told of marvelous cures, to inquire pointedly, "Is that person perfectly well to-day?" The general answer will be, died of something else, or has some other disease.

